# 15d-PGJ2 induces apoptosis of mouse oligodendrocyte precursor cells

**DOI:** 10.1186/1742-2094-4-18

**Published:** 2007-07-16

**Authors:** Zhongmin Xiang, Tong Lin, Steven A Reeves

**Affiliations:** 1 CNS Signaling Laboratory, MassGeneral Institute for Neurodegenerative Disease (MIND), Massachusetts General Hospital, Harvard Medical School, 114 16^th ^Street, Charlestown, MA 02129, USA

## Abstract

**Background:**

Prostaglandin (PG) production is associated with inflammation, a major feature in multiple sclerosis (MS) that is characterized by the loss of myelinating oligodendrocytes in the CNS. While PGs have been shown to have relevance in MS, it has not been determined whether PGs have a direct effect on cells within the oligodendrocyte lineage.

**Methods:**

Undifferentiated or differentiated mouse oligodendrocyte precursor (mOP) cells were treated with PGE2, PGF2α, PGD2 or 15-deoxy-^Δ12,14^-PGJ2 (15d-PGJ2). Cell growth and survival following treatment were examined using cytotoxicity assays and apoptosis criteria. The membrane receptors for PGD2 and the nuclear receptor peroxisome proliferator-activated receptor (PPAR)γ, as well as reactive oxygen species (ROS) in the death mechanism were examined.

**Results:**

PGE2 and PGF2α had minimal effects on the growth and survival of mOP cells. In contrast, PGD2 and 15d-PGJ2 induced apoptosis of undifferentiated mOP cells at relatively low micromolar concentrations. 15d-PGJ2 was less toxic to differentiated mOP cells. Apoptosis was independent of membrane receptors for PGD2 and the nuclear receptor PPARγ. The cytotoxicity of 15d-PGJ2 was associated with the production of ROS and was inversely related to intracellular glutathione (GSH) levels. However, the cytotoxicity of 15d-PGJ2 was not decreased by the free radical scavengers ascorbic acid or α-tocopherol.

**Conclusion:**

Taken together, these results demonstrated that 15d-PGJ2 is toxic to early stage OP cells, suggesting that 15d-PGJ2 may represent a deleterious factor in the natural remyelination process in MS.

## Background

Prostaglandin (PG)s are a group of 20-carbon fatty acids derived from membrane lipids. By sequential enzymatic reactions of phospholipase A2 (PLA2), housekeeping cyclooxygenase (COX)-1 or inducible COX-2, PGH2 is generated and then converted to PGE2, PGD2, PGF2α, PGI2 (prostacyclin) and TXA2 (thromboxane A2) by their respective PG isomerases [1]. For example, PGH2 is first converted to PGD2 by lipocalin-type PGD2 synthase (L-PGDS) or hematopoietic (H)-PGDS, which then undergoes sequential non-enzymatic dehydration reactions to form 15-deoxy-^Δ12,14^-PGJ2 (15d-PGJ2). PGs generally act through membrane-bound G-protein coupled PG receptors with the exception of 15d-PGJ2, which has no defined membrane receptor, although reported to be an activator of the PGD2 receptor DP2 [2]. Instead, 15d-PGJ2 is a natural ligand for the nuclear receptor peroxisome proliferator-activated receptor (PPAR)γ [3], which has a major role in the regulation of proliferation, differentiation and lipid metabolism [4,5]. Moreover, 15d-PGJ2 has been shown to induce apoptosis of cultured cortical neurons [6,7], endothelial cells [8], hepatic myofibroblasts [9], granulocytes [10] and cancer cells [11], through both PPARγ-dependent and PPARγ-independent mechanisms [9,10].

Mounting evidence suggests that PGs play important roles in neuroinflammatory diseases such as multiple sclerosis (MS), an autoimmune disease of the central nervous system (CNS) in which T- and B cells attack components of the myelin sheath leading to loss of myelin as well as myelinating oligodendrocytes [12-14]. As a natural repair mechanism, oligodendrocyte precursor (OP) cells proliferate and differentiate within the demyelination sites to replenish the lost myelinating oligodendrocytes [15,16]. In patients with MS and in the experimental autoimmune encephalomyelitis (EAE) rodent model, the demyelination foci are typically characterized by inflammatory infiltrates containing myelin-specific T- and B cells, and activated microglia and astrocytes [12,14,17-19]. These inflammatory cells are known to secrete cytotoxic cytokines such as TNFα and interleukin (IL)-6 [12,20], as well as PGs such as PGE2, PGD2 and PGF2α [21-23]. Bacterial lipopolysaccharide (LPS), which is a potent proinflammatory factor that induces abundant PGD2 or 15d-PGJ2 production in microglia cultures [24,25], and in the CSF and spinal cord following systemic administration [26,27]. In MS demyelination foci, gene expression of PG related enzymes such as PLA2 [28], COX-2 [29] and L-PGDS [30] are up-regulated. Increased L-PGDS in peri-neuronal oligodendrocytes and H-PGDS in microglia are also observed in the mouse *twitcher *demyelination model [31,32]. Additional evidence has shown that H-PGDS is increased in activated T helper (Th)2 cells *in vitro *[23]. While these findings suggest that OP cells are exposed to a PG-rich environment, little is known regarding the effect these PGs have on OP cells.

In this study, we examined the effect of PGs on mouse OP (mOP) cells. We found that PGD2 and its dehydration end product 15d-PGJ2 induce apoptosis of OP cells in a PPARγ-independent manner, while more mature OP cells are relatively resistant. These results suggest that PGD2 and 15d-PGJ2 may contribute to MS pathology by inducing OP cell death.

## Methods

### Materials and reagents

N1 supplement, insulin, biotin, staurosporine, indomethacin, NS398, SC58125, GW9662, N-acetyl cysteine (NAC), buthionine sulfoximine (BSO), ascorbic acid, α-tocopherol, poly-D-lysine, 3-(4,5-dimethylthiazol-2-yl)-2,5-diphenyltetrazolium bromide (MTT) and bisbenzimide were obtained from Sigma (St. Louis, MO); High glucose DMEM, DMEM/F12 (1:1), fetal bovine serum, penicillin/streptomycin, Trizol, PCR reagents and enzymes were from Invitrogen (Carlsbad, CA); SYBR green PCR mix was from Amersham (Piscataway, NJ); 15d-PGJ2, PGD2, PGE2, PGF2α, T0070907, AH6809, BAY-u3405 and GSH kit were from Cayman Chemicals (Ann Arbor, MI); Cover-slips were from Bellco Biotechnology (Vineland, NJ); LDH cytotoxicity assay kit was from Promega (Madison, WI); TUNEL kit and cell death ELISA kit were from Roche (Indianapolis, IN); Fluorescence probe 5-(and-6)-carboxy-2',7'-dichlorodihydrofluorescein diacetate (carboxy-H_2_DCFDA) was from Molecular Probes (Eugene, OR); Goat anti-MBP was from Santa Cruz Biotechnology (Santa Cruz, CA); rabbit anti-NG2 was kindly provided by Dr. W. Stallcup; rabbit anti-πGST was from MBL (Woburn, MA); A2B5 hybridoma was from ATCC (Menassas, VA); normal donkey serum and all secondary antibodies were from Jackson ImmunoResearch (West Grove, PA); Fluorescent mounting medium with or without nuclear dye DAPI was from Vector Laboratories (Burlingame, CA).

### Mouse oligodendrocyte precursor (mOP) cell line

The mOP cell line developed in this lab [33] and the rat oligodendrocyte cell line CG4 [34] were used in this study. Both cell lines were maintained in CG4 proliferation medium (PM) as described previously [34]. CG4 PM consists of 70% high glucose DMEM, 30% conditioned medium from B104 neuroblastoma cell line, supplemented with 0.5% N1 supplements, biotin 10 μg/ml, insulin 5 μg/ml and 1% penicillin/streptomycin.

Differentiation of mOP cells was induced in differentiation medium (DM), which is different from CG4 PM only in that the 30% conditioned medium was from confluent mOP cell cultures instead of B104 neuroblastoma cultures. The use of conditioned medium from confluent mOP cells was based on the previous report that oligodendrocytes are self-inhibiting in proliferation [34] and our observation of a differentiation-promoting effect from medium obtained from confluent mOP cell cultures (data not shown). Conditioned medium from confluent cells was obtained as follows: Approximately 50% confluent mOP cell cultures were grown for 1 wk in PM without medium change, medium was collected, filtered, and then used to make DM. mOP cells were cultured in DM for 3 d before treatments.

### Drug treatment of cell cultures

mOP cell cultures were grown to 60–70% confluency in 12- or 24-well plates and then serum-starved (CG4 PM without conditioned medium) for 24 h before experiments. PGs were added to the medium for 24–48 h. For 15d-PGJ2 or PGD2 preparation, the original solvent ethyl acetate was evaporated, and PGD2 or 15d-PGJ2 was re-dissolved in PBS before adding to the medium. For other chemicals, a corresponding amount of the solvent (DMSO or ethanol) was added to control cultures with concentrations less than 0.2%. All experiments were performed 3–5 times and each treatment in triplicates.

### Cell growth/viability assay

Cells were assayed using 3-(4,5-dimethylthiazol-2-yl)-2,5-diphenyltetrazolium bromide (MTT). MTT is converted to a blue formazan product by mitochondria dehydrogenases only in live cells, and can be used as a cytotoxicity assay [35]. In this regard, the MTT assay has been used to specifically address 15d-PGJ2-induced cell death in neurons and endothelial cells [7,8]. Cells were incubated in medium with MTT (50 μg/ml) for 1 h at 37°C. The formazan product was dissolved in DMSO, and absorbance at 600 nm was measured using a plate reader. Additionally, lactate dehydrogenase (LDH) enzymatic activity in the medium was measured using the CytoTox96 kit (Promega) according to the manufacturer's instructions. LDH is released into the medium upon cell lysis, and the activity measured in the medium is therefore proportional to the number of lysed cells. The amount of cell death (percentage) was calculated as released LDH/total LDH (value obtained by lysing all cells in the untreated wells).

### Terminal deoxynucleotidyl transferase (TdT) dUTP nick end labeling (TUNEL) and nuclear staining

TUNEL staining was performed using a kit from Roche following the manufacturer's instructions. In brief, cells that had been grown on coverslips were fixed in 4% paraformaldehyde for 20 min and then rinsed in PBS. After permeablization for 15 min at RT with 0.1% Triton X-I00 in 0.1% citrate buffer, the cells were incubated with TUNEL mix (TdT enzyme and fluorescein-dUTP) for 1 h at 37°C. After rinsing with PBS, the coverslips were mounted on glass slides with fluorescence mounting medium and inspected under a fluorescence microscope. Four random areas for each coverslip (20× objective view) were surveyed and the number of cells counted. For nuclear staining, bisbenzimide was added to the medium at 1 μg/ml for 20 min. After washing, mOP cells were mounted for fluorescence microscopy.

### ELISA-based cell death assay

Apoptotic cell death was also quantified using an ELISA kit that quantifies indirectly the histone-containing nucleosomes after DNA fragmentation. The culture medium was collected. Attached cells were then collected using trypsin digestion (0.25% for 5 min), combined with the culture supernatant, and then the mix was pelleted at 1,500 × g for 5 min. After carefully removing the supernatant, the cells were lysed in incubation buffer for 30 min at RT. After centrifugation at 20,000 × g for 10 min, the supernatants (cytoplasmic fraction containing nucleosomes) were added to the plate according to the manufacturer's instructions. DNA fragmentation was then examined calorimetrically using a plate reader at 405 nm.

### DNA gel electrophoresis

Cells were harvested and lysed in hypotonic buffer (50 mM Tris (pH7.9) containing 1% Triton X-I00, 10 mM EDTA and 50 μg/ml RNase A) for 5 min at RT. The lysates were centrifuged at 10,000 × g for 10 min, and the supernatant containing short DNA fragments was collected. After phenol/chloroform extraction, DNA was precipitated with sodium acetate and ethanol, resuspended in TE buffer, separated on a 1.2% agarose gel containing ethidium bromide and then visualized with a UV illuminator.

### Immunocytochemistry

mOP cells grown on poly-D-lysine coated cover-slips were fixed in 4% paraformaldehyde for 20 min and rinsed in PBS. After permeablization for 15 min at RT with 0.2% Triton X-I00 in PBS and 10% normal donkey serum to block unspecific binding, mOP cells were incubated with primary antibodies: goat anti-MBP (1: 100), rabbit anti-NG2 (1: 200), rabbit anti-GST (1: 1000), all diluted in PBS with 1% normal donkey serum. For A2B5, hybridoma medium was used directly without dilution. After three washes with PBS, the cells were incubated with appropriate Cy2- or Cy3-conjugated secondary antibodies (1:200 in PBS with 1% donkey serum) for 1 h at RT in the dark. After PBS washes, the cover-slips were mounted on slides with fluorescence mounting medium containing the nuclear dye DAPI and examined using an Olympus BX60 microscope equipped with epifluorescence optics.

### Reactive oxygen species (ROS) detection

ROS production was detected using the fluorescence probe carboxy-H_2_DCFDA. mOP cells plated on poly-D-lysine coated coverslips were washed twice with DMEM and then incubated in loading solution (DMEM with 25 μM DCFDA) for 30 min at 37°C in the dark. Cells were washed twice and then treated with 15d-PGJ2. Coverslips were rinsed with DMEM before mounting on slides and fluorescence (FITC filter) images of cells were taken immediately using a fluorescence microscope equipped with a digital camera (DP70, Sony). Ten fields (40× objective) for each coverslip were sampled (>400 cells), the mean pixel values (0–255) of individual cells were analyzed using NIH imaging software (NIH, Bethesda, MD). All treatments were performed in duplicate and data expressed were averaged values of all cells counted in each condition.

### GSH measurement

Total intracellular GSH content was measured using a kit from Cayman according to the manufacturer's instructions. In brief, mOP cells were scraped from 6-well plate, pelleted by centrifugation at 700 × g for 5 min, homogenized in 1 ml cold buffer, and then centrifuged at 10,000 × g for 15 min at 4°C. The supernatant was collected and protein concentration was measured. The supernatant was deproteinated by mixing with metaphosphoric acid before GSH content measurement. GSH content was expressed as μmol/mg protein.

### RT-PCR

Total RNA was isolated using the Trizol reagent according to the manufacturer's instructions. First-strand cDNA was synthesized using reverse transcriptase (Superscript) and oligo(dT) primer. PCR reactions were performed using 1 μg cDNA and Taq polymerase. Primers for PPARγ amplification were: 5'-TTT TCA AGG GTG CCA GTT TC-3' and 5'-AAT CCT TGG CCC TCT GAG AT-3'. The expected PCR product size is 198 bp. All reactions were carried out with iCycler (BioRad, Hercules, CA) using SYBR green PCR mix, which allows automated signal quantification. The PCR parameters were 35 cycles with 94°C denaturation for 20 sec, 60°C annealing for 30 sec, and 72°C extension for 50 sec. Quantification was performed using the ΔΔ method. The PCR products were confirmed by ethidium bromide-stained agarose gel electrophoresis. cDNA derived from a postnatal day 20 mouse brain was used as a positive control.

### Statistics

Statistical analysis was performed using InStat and Prism software (GraphPad Software, San Diego, CA). Student t-test (two-tailed) was used to assess the difference between two groups. One-way ANOVA was used to assess differences among groups (more than three) with Newman-Keuls post-test. When appropriate, two-way ANOVA and Bonferroni posttest were used to assess differences among groups with two independent variables. All significance levels were set at p < 0.05.

## Results

### PGD2 and 15d-PGJ2 but not PGE2 or PGF2α induced mOP cell death

**Figure 1:**
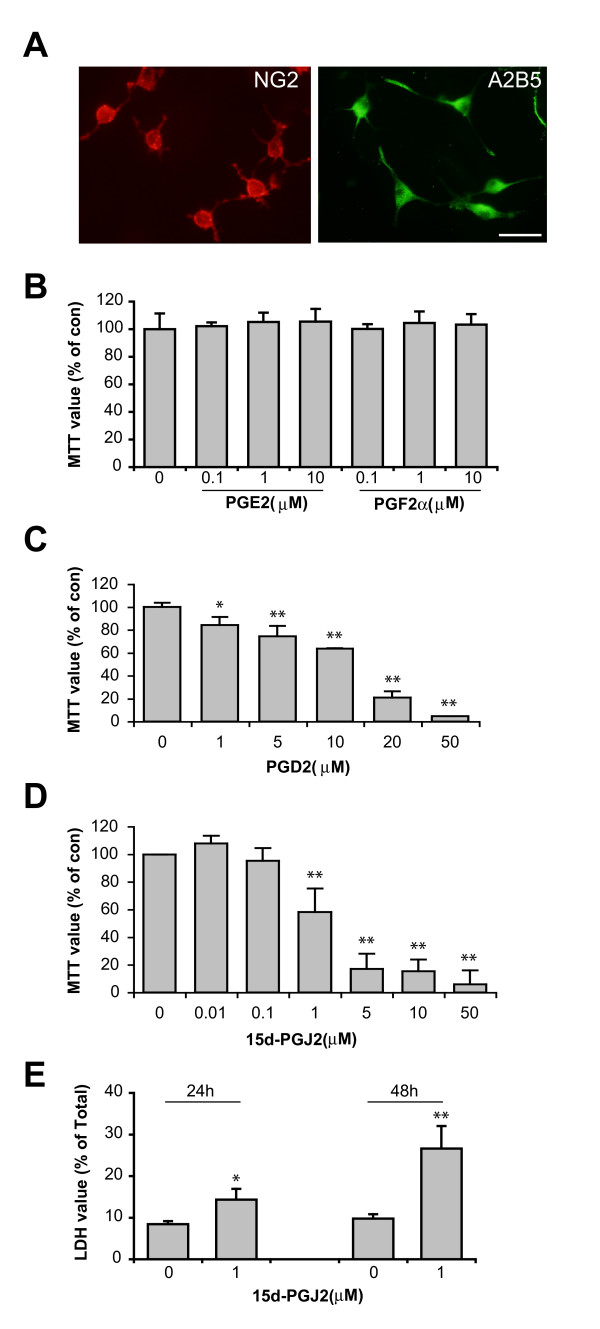
**The effect of PGs on the growth and survival of mouse oligodendrocyte precursor (mOP) cells**. (A) mOP cells express the oligodendrocyte precursor surface markers NG2 (red) and A2B5 (Green). Scale bar, 20 μm. (B) mOP cells were treated with PGE2 or PGF2α (0.1, 1 and 10 μM) and examined using the MTT assay after 24 h. (C-D) mOP cells were treated with the indicated concentrations of PGD2 or 15d-PGJ2 and examined using the MTT assay after 24 h. Data are the average of 3–4 experiments and expressed as percentage of the control group (vehicle treated). (E) mOP cells were treated with 15d-PGJ2 (1 μM) and examined using the LDH assay after 24 h and 48 h. Data are expressed as percentage of the total LDH. Asterisks indicate significant difference versus control group (One-way ANOVA with Dunnet posttest for C and D, Student t-test for E, *(p < 0.05), **(p < 0.01).

We have previously developed mouse OP (mOP) cells [33] from the post-natal mouse brain that can be sustained for long periods in culture and which display properties similar to those of the rat CG4 oligodendrocyte cell line [34]. When grown in proliferation medium (PM) mOP cells assume bipolar or tripolar morphology and express the OP cell markers NG2 and A2B5 (Fig. 1A). We first examined whether endogenous PG production by oligodendrocytes has a role in mOP cell growth and survival. In these experiments we used MTT assay as an initial assay for cell death and chemical inhibitors of enzymes responsible for PG production. mOP cells were treated with 10 μM of indomethacin (COX-1 and COX-2 inhibitors), NS398 (COX-2 specific inhibitor) or SC58125 (COX-2 specific inhibitor) for 48 h. No differences in mOP cell growth and survival were observed compared to vehicle treated (Data not shown). We next tested whether direct applications of PGs to the culture medium of mOP cells affected growth and survival. PGE2 or PGF2α treatment (0.1, 1 and 10 μM) for 24 h had no effect on mOP cell growth (Fig. 1B). Extended treatment with PGE2 or PGF2α (10 μM) for 48 h also had no effect (data not shown). In contrast, PGD2 and 15d-PGJ2 induced significant cell death in a dose-dependent manner as early as 24 h (Fig. 1C–D). mOP cells were more sensitive to 15d-PGJ2 (50% effective concentration (EC50) 1.0 μM) than to PGD2 (EC50 16.6 μM). To confirm the cytotoxicity of 15d-PGJ2, we used a more specific cell death assay, which measures the enzymatic activity of lactate dehydrogenase (LDH) released in the medium by dead cells. Treatment of 15d-PGJ2 (1.0 μM) induced significant cell death at 24 h, and more dramatically at 48 h. The effect of these PGs was also examined on the rat oligodendrocyte cell line CG4 using MTT assay and similar results were observed (data not shown).

### Apoptotic death of mOP cells induced by 15d-PGJ2

**Figure 2:**
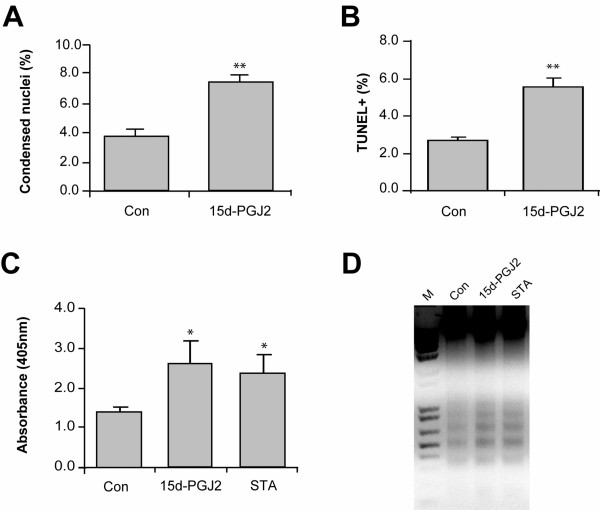
**Characterization of 15d-PGJ2-induced apoptosis in mOP cells**. mOP cells were treated with 15d-PGJ2 (1 μM) for 24 h, and then stained with the nuclear dye bisbenzimide or labeled with the TUNEL method. Cells with condensed nuclei (A) or that were TUNEL-positive (B) were counted, and expressed as percentage of the total number of cells. For apoptosis ELISA (C) and DNA fragmentation gel analysis (D), DNA from mOP cells that had been treated with 15d-PGJ2 (5 μM) for 24 h was extracted and analyzed. Staurosporine (STA, 100 nM) treatment was used as positive control. M, molecular standards. Asterisks indicate significant difference versus control group (t-test, two-tailed, *(p < 0.05), **(p < 0.01)).

We next examined whether mOP cell death induced by 15d-PGJ2 was apoptotic. mOP cells were treated with 15d-PGJ2 for 24 h and assayed for apoptosis by staining with the DNA-binding dye bisbenzimide, which can demonstrate nuclear condensation characteristic of apoptosis, and using the TUNEL staining method, which detects apoptosis-associated DNA strand breaks [36]. In untreated mOP cells, a small percentage (~3.8%) of the cells displayed condensed nuclei when stained with bisbenzimide (Fig. 2A). However, when mOP cells were treated with 1 μM 15d-PGJ2 for 24 h the percentage of cells with condensed nuclei doubled to 7.5% (Fig. 2A). TUNEL staining revealed similar results where 2.5% of untreated and 6.0% PGJ2-treated cells were positive for TUNEL staining (Fig. 2B). Further evidence for 15d-PGJ2 induced apoptosis of mOP cells was obtained using an ELISA-based cell death assay, which quantifies indirectly the histone-containing nucleosomes generated due to DNA fragmentation. In this assay, 15d-PGJ2 induced apoptotic DNA fragmentation ~2-fold over that observed in untreated cells (Fig. 2C). Lastly, we assessed DNA fragmentation (mono- and oligonucleosomes) in 15d-PGJ2-treated mOP cells using agarose gel electrophoresis. 15d-PGJ2 increased DNA fragmentation in mOP cells over that observed in untreated cells (Fig. 2D). Staurosporine (STA), a well described inducer of apoptosis [37], induced DNA fragmentation in mOP cells similar to that of 15d-PGJ2-treated cells (Fig. 2C–D).

### 15d-PGJ2-induced apoptosis of mOP cells occurs independently of PPARγ or PGD2 receptors

**Figure 3:**
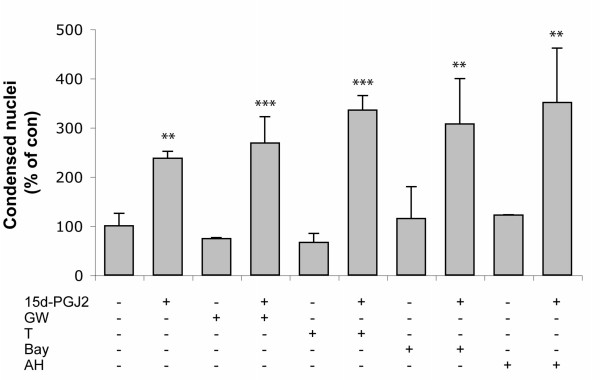
**15d-PGJ2 cytotoxicity on mOP cells occurs independently of PPARγ or PGD2 membrane receptors**. mOP cells cultured on coverslips were treated with 15d-PGJ2 (1 μM), in the absence or presence of the irreversible PPARγ antagonists GW9662 (GW, 10 μM) or T0070907 (T, 100 nM), or nonspecific PGD2 receptor (DP) antagonist AH6809 (AH, 10 μM) or specific DP2 antagonist BAY-u3405 (BAY, 5 μM) for 24 h, and apoptotic cell death was examined using bisbenzimide staining. Cells were counted and expressed as percentage of control (untreated). Data shown were average of three experiments. Asterisks indicate significant difference versus control group (Two-way ANOVA Bonferroni posttest, **(p < 0.01), ***(p < 0.001)).

15d-PGJ2 is a natural ligand for PPARγ and has been shown to induce apoptosis in a variety of cell types through a PPARγ-dependent pathway [6,8]. We therefore investigated whether 15d-PGJ2-induced apoptosis in mOP cells was through a PPARγ-dependent pathway. Real time RT-PCR analysis demonstrated a small amount of PPARγ amplification in mOP cells (data not shown). To further examine whether PPARγ has a role in 15d-PGJ2 induced apoptosis of mOP cells we tested whether pharmacological inhibition of PPARγ protects mOP cells from the cytotoxic effects of 15d-PGJ2. Pre-incubation of mOP cells with the irreversible PPARγ antagonists GW9662 (10 μM) or T0070907 (100 nM) did not block 15d-PGJ2-induced apoptotic cell death (Fig. 3). These results provide evidence that the cytotoxic effect of 15d-PGJ2 is not mediated through the PPARγ pathway.

15d-PGJ2 has been reported to be an activator of the G-protein-coupled receptor PGD2 DP2 [2]. To test whether 15d-PGJ2 exerts its effect through the PGD2 DP2 receptor, mOP cells were pretreated with the non-specific DP antagonist AH6809 (which blocks both DP1 and DP2 receptors) or the specific PGD2 DP2 receptor antagonist BAY-u3405. Neither BAY-u3405 (5 μM) nor AH6809 (10 μM) blocked 15d-PGJ2-induced death (Fig. 3). These results suggest that 15d-PGJ2 induces mOP cell death independently of known membrane G-protein-coupled receptors for PGD2.

### 15d-PGJ2 cytotoxicity and ROS

**Figure 4:**
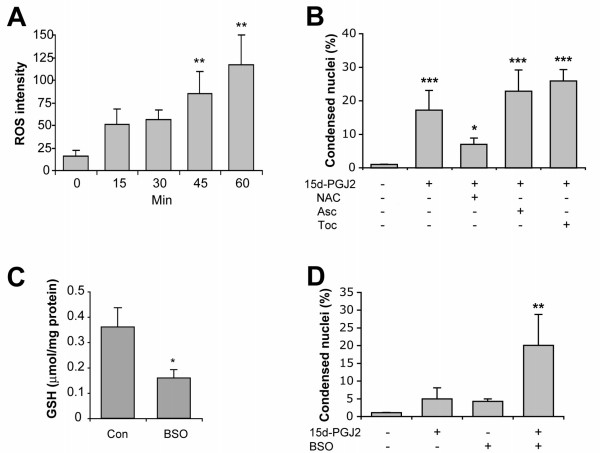
**15d-PGJ2 cytotoxicity involves free radical production and is influenced by intracellular glutathione levels**. (A) Time course of 15d-PGJ2-induced ROS production. mOP cells were preloaded with the fluorescent ROS probe DCFDA for 30 min, and then treated with 15d-PGJ2 (10 μM) for 1 h. ROS production was expressed as DCFDA fluorescence intensity (pixel value). (B) mOP cells were pre-treated or not with NAC (1 mM), Ascorbic acid (1 mM) or α-tocopherol (1 mM) for 1 h prior to treatment of 15d-PGJ2 (5 μM) for 24 h, toxicity was examined by counting the apoptotic cells with condensed nuclei. (C) mOP cells were treated or not with BSO (100 μM) for 4 h and the total level of intracellular GSH was measured. (D) mOP cells were treated with BSO (100 μM) for 1 h and then co-treated with 15d-PGJ2 (1 μM) for 24 h. Cells treated with BSO or 15d-PGJ2 alone or untreated were included as controls. Toxicity was examined by counting the apoptotic cells with condensed nuclei. Asterisks indicate significant difference (One-way ANOVA with Newman-Keuls or Dunnet posttest, or two way ANOVA with Bonferroni posttest, *(p < 0.05), **(p < 0.01) ***(p < 0.001); two-tailed t-test used in C).

Previous reports have suggested that 15d-PGJ2 may induce intracellular oxidative stress [38,39]. To examine whether there is increased ROS production in 15d-PGJ2-treated mOP cells we preloaded cells with the fluorescent ROS probe DCFDA prior to 15d-PGJ2-treatment. ROS production was significantly increased in mOP cells as early as 45 min after treatment with 15d-PGJ2 (10 μM) (Fig. 4A). Glutathione (GSH) is an important antioxidant that protects cells from oxidative damage by ROS. We therefore tested whether manipulations of the intracellular level of GSH could affect apoptotic cell death induced by 15d-PGJ2. Pretreatment of mOP cells with NAC, which is a precursor molecule for GSH synthesis and a reducing agent for oxidized GSH [40], provided ~60% protection against 15d-PGJ2 induced death, while pre-incubation with the antioxidants ascorbic acid or α-tocopherol did not provide protection (Fig. 4B). In contrast, application of buthionine sulfoximine (BSO), an inhibitor for γ-glutamylcysteinase synthatase [41] which depletes intracellular GSH (Fig. 4C), was toxic to mOP cells by itself and sensitized mOP cells to a lower concentration of 15d-PGJ2 (Fig. 4D). These results suggest that the toxicity of 15d-PGJ2 to mOP cells is related to intracellular GSH levels.

### 15d-PGJ2 cytotoxicity is dependent on the stage of oligodendrocyte maturation

**Figure 5:**
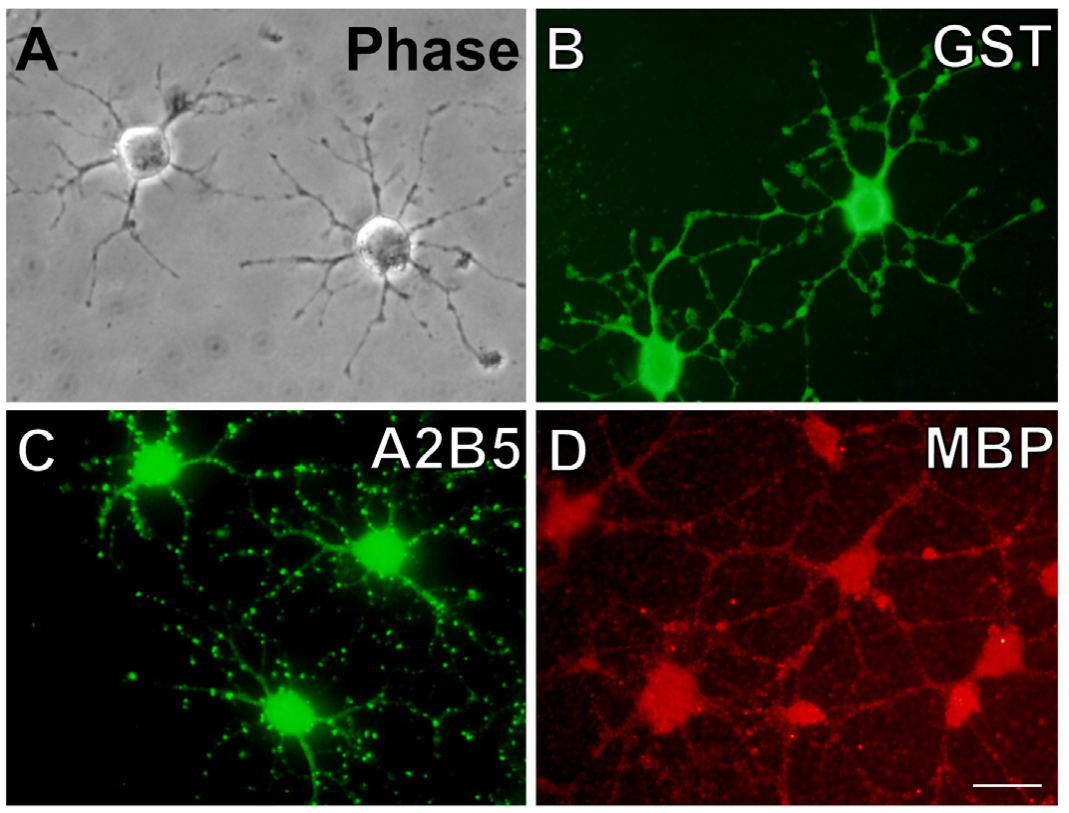
**Micrographs showing mOP cell differentiation**. mOP cells were induced to differentiate in DM. Differentiated mOP cells display elaborate process extension with secondary and tertiary branching (A, Phase), and immunostain positive for the mature oligodendrocyte markers πGST (B, Green) and MBP (D, Red). However, differentiated mOP cells still stain positive for the early stage OP cell marker A2B5, in a punctate fashion (C, Green). Scale bar, 20 μm.

Developmental stage susceptibility to various cytotoxic stimuli has been reported previously [42-45]. We tested whether the effect of 15d-PGJ2 on oligodendrocytes is stage-dependent. mOP cells were induced to differentiate in differentiation medium (DM). In contrast to the simple morphology that undifferentiated mOP cells display (97.7% with 3 or less processes and 2.3% with 4 to 6 branches, n = 287 cells) (Fig. 1A), differentiated mOP cells display complex process formation (17.9% with 3 or less processes, 50.2% with 4 to 6 branches, and 31.9% with more than 6 branches, n = 304 cells). While displaying increased immunoreactivity to the late stage markers π-GST and MBP, differentiated mOP cells still showed punctate staining to early stage OP cell marker A2B5 (Fig. 5A–D).

**Figure 6:**
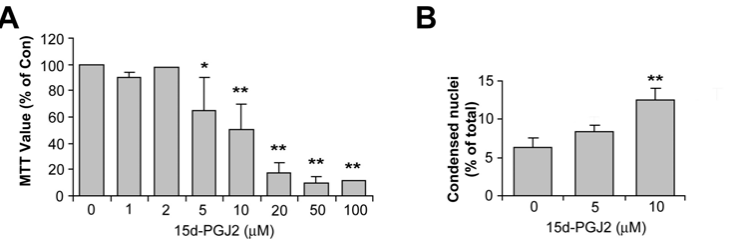
**The effect of 15d-PGJ2-induced death is dependent on the developmental stage of oligodendrocytes**. mOP cells were induced to differentiate in DM and then treated with 15d-PGJ2 for 24 h. Toxicity was examined by MTT assay (A) and by counting cells with condensed nuclei stained by the nuclear dye bisbenzimide (B). Asterisks indicate significant difference (One-way ANOVA with Dunnet post test, *(p < 0.05), **(p < 0.01).

When differentiated mOP cells were treated with 15d-PGJ2, higher concentrations of 15d-PGJ2 were needed for significant cytotoxicity as judged using MTT assay (Fig. 6A), with an EC50 of 9.8 μM. Maturation stage-specific cytoxicity was also measured using apoptosis criteria (condensed nuclei). While 1 μM 15d-PGJ2 was sufficient to induce significant cell death of undifferentiated mOP cells (see Fig. 2A), a 10-fold higher concentration of 15d-PGJ2 was required to induce comparable death in differentiated mOP cells (Fig. 6B). These results demonstrated that differentiated mOP cells are more resistant to 15d-PGJ2-induced cytotoxicity.

## Discussion

While PGs have been shown to display a range of activities on various cell types [1], few studies have been carried out on cells within the oligodendrocyte lineage. Our data indicate that PGD2/15d-PGJ2 may represent another group of factors in addition to cytotoxic cytokines produced during inflammation that are toxic to OP cells.

### PG production

Our results demonstrated that 15d-PGJ2 at ≥1 μM is toxic to mouse OP cells. While baseline production of PGD2 from the whole mouse brain has been calculated to be approximately 2 nM [46], higher concentrations may, however, occur during inflammatory conditions. LPS treatment can mimic inflammatory conditions and induces PGD2/15d-PGJ2 production in mixed glial cell cultures [24,25] and in animal models [26,27]. The production of 15d-PGJ2 in the medium of primary microglial cell cultures was calculated to be in the range of 10 nM [25]. However, it is likely that 15d-PGJ2 concentrations *in vivo *can be orders higher because of the more confined interstitial space. Indeed this has been observed previously for extracellular levels of glutamate. Upon inhibition of glutamate uptake the interstitial glutamate concentration increases to over 100–150 times (200–300 nM) the minimal value maintainable by glutamate transporters (2 nM) [47]. In a more recent study, 15d-PGJ2 was found to be increased to 600 pg/mg protein [48] (~0.1 μM), in the ischemic cortex. These results suggest that the toxic levels of 15d-PGJ2 we observed in our *in vitro *experiments may also occur *in vivo*.

### Death mechanism

Our results demonstrated that 15d-PGJ2 at 1 μM decreases MTT values at 24 h by ~50%. This relatively large reduction likely represents a combination of cell death, reduced cell proliferation, and compromised mitochondrial activity. Using a more cell death specific LDH assay, and an array of apoptotic assays, we demonstrated that 15d-PGJ2 induces apoptotic cell death in mOP cells, which has been observed in other cell types [6-11].

The mechanism(s) for this apoptosis has not been clearly elucidated. While 15d-PGJ2 is a known ligand for PPARγ and has been implicated in apoptosis in a variety of cell types [6,8], in our studies 15d-PGJ2 induced apoptotic death of mOP cells independently of PPARγ, since the irreversible PPARγ antagonists GW9662 or T0070907 did not provide protection. Consistent with our findings, 15d-PGJ2 toxicity is observed in hepatic myofibroblasts that lack PPARγ expression [9].

15d-PGJ2 has been shown previously to induce free radical production [38,39], potentially due to its unsaturated α, β carbonyl moieties in the cyclopentanone rings. In addition, these moieties are capable of reacting with thiol groups by Michael addition [49] and thus able to modify the functions of important proteins such as thioredoxin [39]. Reduced GSH is the most abundant non-protein thiol group-containing molecule and can readily conjugate to 15d-PGJ2 via glutathione-S-transferase (GST). Conjugation prevents free 15d-PGJ2 from attacking other intracellular targets. In this regard, our studies show that depleting intracellular GSH by BSO potentiates 15d-PGJ2-induced cytotoxicity and increasing intracellular levels of reduced GSH (by NAC) provides protection. In our study, antioxidants such as ascorbic acid or α-tocopherol, which act as electron donors to halt free radical production, provided no protection for the cytotoxic effect of 15d-PGJ2 on mOP cells. This has been observed previously in neuronal cell types where ascorbic acid did not provide protection against PGJ2-induced toxicity [50], or dopamine-induced apoptosis [51]. This lack of protection may be due to ascorbic acid-mediated depletion of intracellular GSH pool which would offset its beneficial effect [50]. Interestingly, in a study using cultured OP cells, ascorbic acid provided no protection against cystine deprivation-induced death, while α-tocopherol provided protection, without blocking the depletion of intracellular GSH [42]. Taken together with our results these findings suggest that ROS production may not be the only event responsible for 15d-PGJ2-induced cell death. These other events may include reduction in mitochondrial membrane potential [52], inhibition of NFκB activation [53], and inhibition of transcription factor AP-1 associated [54] gene expression that is involved in cell survival and apoptosis [1,55].

Of special interest, our studies demonstrate that 15d-PGJ2 is more toxic to early stage OP cells than to their more differentiated counterpart. The higher resistance that mature oligodendrocytes display has been reported previously in response to lysophosphatidic acid [45], IFNγ [43], cysteine deprivation and hydrogen peroxide (H_2_O_2_) treatment [42,44]. Although the death mechanisms may be different, they all include oxidative stress and suggest that mature oligodendrocytes have a better system to fend off oxidative stress. While GSH may be more directly involved in providing protection against oxidative stress, mature oligodendrocytes do not in fact have a higher GSH level than immature oligodendrocytes [42]. In this regard, maturational up-regulation of glutathione peroxidase [44], π-glutathione-S-transferase [56] and L-PGDS (also a GST) [57] in oligodendrocytes, may contribute to more effective removal of electrophilic molecules such as 15d-PGJ2.

### Perspective

While our studies has demonstrated that 15d-PGJ2 is cytotoxic to OP cells, we are aware that 15d-PGJ2 can have effects on other cells that contribute to the demyelination and remyelination process. Several previous cell culture studies have shown that 15d-PGJ2 can inhibit activation of microglia [25,58,59] and astrocytes [60], and is toxic to microglia depending on its concentration [25]. Moreover, systemic application of 15d-PGJ2 has been shown to be protective in the rodent EAE model [61-63], which is consistent with its inhibitory effect on microglia and immune cells. However, the beneficial effect due to microglia inhibition and toxic effect on OP cells are not mutually exclusive. The pathological role of 15d-PGJ2 *in vivo*, therefore, remains to be better defined and likely will depend on the level of endogenous 15d-PGJ2 production. Further investigations, taking into consideration the interaction between producer and recipient cells in a defined brain area, are clearly warranted to elucidate the role of 15d-PGJ2 *in vivo*.

## Conclusion

In conclusion, we found that PGE2 and PGF2α have minimal effects on the growth and survival of mOP cells, while PGD2 and 15d-PGJ2 induce apoptosis at low micromolar concentrations independently of membrane receptors for PGD2 and the nuclear receptor PPARγ. The cytotoxicity of 15d-PGJ2 on mOP cells is associated with the production of ROS, and affected by manipulations of intracellular glutathione level but not by the free radical scavengers ascorbic acid or α-tocopherol. Additionally, 15d-PGJ2 is more toxic to early stage OP cells than to differentiated OP cells. Taken together, these results suggest that 15d-PGJ2 may represent a deleterious factor in the natural remyelination process in MS.

## Competing interests

The author(s) declare that they have no competing interests.

## Authors' contributions

ZX and TL performed the experiments. ZX and SAR conceived the project and drafted the manuscript. All authors have read and approved the final version.
